# Factors Affecting Visual Outcome After Traumatic Cataract Surgery in a Tertiary Hospital in Ahvaz, Iran

**DOI:** 10.18502/jovr.v21.18169

**Published:** 2026-07-20

**Authors:** Mahmoodreza Panahibazzaz, Hani Saber Moghadam, Nasrin Masihpour, Farshad Ostadian, Elham Farhadi, Maryam Adabi, Sadid Hooshmandi

**Affiliations:** ^1^Department of Ophthalmology, Infectious Ophthalmologic Research Center, Ahvaz Jondishapur University of Medical Sciences, Ahvaz, Iran; ^2^Clinical Research Development Unit, Golestan Hospital, Ahvaz Jondishapur University of Medical Sciences, Ahvaz, Iran; ^3^Research Center for Food Hygiene and Safety, School of Public Health, Shahid Sadoughi University of Medical Sciences, Yazd, Iran; ^4^Ophthalmic Research Center, Research Institute for Ophthalmology and Vision Science, Shahid Beheshti University of Medical Sciences, Tehran, Iran

**Keywords:** Cataract, Surgery, Visual Outcome

## Abstract

**Purpose:**

Ocular trauma represents a considerable etiological factor for unilateral blindness, especially in developing countries. This research was conducted to examine the determinants influencing visual acuity after surgical intervention for traumatic cataracts.

**Methods:**

This retrospective study investigated individuals diagnosed with traumatic cataract who were referred and subsequently underwent surgical intervention in a tertiary hospital in Ahvaz, Iran, from June 2013 to October 2023. All patients were contacted, and a thorough ophthalmological evaluation was performed for each person. Visual acuity was assessed utilizing a range of techniques, including central, steady, maintained (CSM) fixation, LEA symbols, Teller acuity cards, and the Snellen chart. Data were analyzed using SPSS version 25.0, and a *P*-value < 0.05 was considered statistically significant.

**Results:**

A total of 64 eyes from 64 patients were included. The mean age was 25.5 ± 22.0 years, and 71.9% were male. Open globe injuries accounted for 75% of cases, most (38%) of which were caused by sharp metal objects. Seventeen patients (26.6%) were under 7 years of age. Significant factors associated with final visual acuity included age (*P* = 0.021), injury type (*P *= 0.025), vitreous prolapse (*P* = 0.012), and size of rupture (*r* = 0.327, *P* = 0.024). Younger patients and those without vitreous prolapse had better visual outcomes.

**Conclusion:**

Age, injury type, vitreous involvement, and wound size were the main predictors of final visual acuity after traumatic cataract surgery. Comprehensive evaluation and timely surgical management may improve visual prognosis.

##  INTRODUCTION

Ocular trauma is a major cause of monocular blindness, particularly in developing countries. One of the most common complications of ocular trauma is cataract formation.^[1]^ Understanding the underlying causes of eye injuries is essential for developing effective preventive strategies, as these injuries often impose a substantial burden on affected individuals and society. This issue becomes even more critical in pediatric cases due to the risk of amblyopia. Studies have shown that between 12% and 46% of all pediatric cataracts are caused by eye trauma. Therefore, allocating financial and healthcare resources based on the local prevalence of traumatic injuries and the high risk of amblyopia in children can help reduce both healthcare expenditures and societal costs.^[2,3]^

Various methods have been established to evaluate visual outcomes in eyes with cataract, whether resulting from trauma or other etiologies.^[4]^ However, concomitant damage to surrounding ocular tissues may complicate surgical outcomes in cases of traumatic cataract.^[5]^ Consequently, prognosis may differ between traumatic and nontraumatic cataracts.^[1]^

Although traumatic cataracts are treatable, their optimal management plays a crucial role in reducing the global burden of blindness. Only a limited number of studies have investigated the factors influencing final visual outcomes after traumatic cataract surgery, and their findings remain inconsistent. Some studies have reported better visual outcomes following surgery in closed globe injuries, whereas others have indicated superior results in open globe injuries.[3,6-8] Given the importance of this topic and the scarcity of research in this area, the present study aimed to identify factors affecting visual acuity following traumatic cataract surgery.

##  METHODS

This retrospective cohort study was conducted in accordance with the Declaration of Helsinki and was approved by the Ethics Committee of Ahvaz Jundishapur University of Medical Sciences (approval number: IR.AJUMS.HGOLESTAN.REC.1403.072). The study included patients with traumatic cataracts who were referred to and underwent surgery at a tertiary hospital in Ahvaz, Iran, between June 2013 and October 2023. The study excluded cases due to the following reasons: lack of consent to participate, failure to attend after the call for participation, follow-up periods shorter than 12 months, surgeries performed by someone other than the designated surgeon (MP), and cases involving complications such as glaucoma, retinal damage, or other issues that led to secondary surgeries or a final visual acuity of light perception (LP) or worse.

All surgeries were performed by a single experienced surgeon specializing in traumatic cataract management (MP). All patients were contacted and underwent a comprehensive ophthalmic examination. Visual acuity was assessed using age-appropriate methods, including central, steady, maintained (CSM) fixation, LEA symbols, Teller acuity cards, or the Snellen chart. Anterior segment evaluation was performed using a slit-lamp biomicroscope, and intraocular pressure was measured by Goldmann applanation tonometry. Pupils were dilated, and posterior segment evaluation was subsequently performed. Preoperative data on the same parameters were retrieved from patients' medical records.

The type of injury was classified as open globe or closed globe based on the Birmingham eye trauma terminology system (BETTS). The etiology of each injury and the anatomical zone involved were recorded. Zone 1 included the cornea up to the limbus; Zone 2 comprised the sclera up to 5 mm beyond the limbus; and Zone 3 referred to injuries extending more than 5 mm posterior to the limbus.

For open globe injuries, the time from trauma to primary repair was considered the initial time point, and the interval between primary repair and cataract surgery was recorded as the secondary time point. For closed globe injuries, the time from trauma to cataract surgery was considered as a single time point. The size of the laceration and injury zone was also documented.

All surgeries were initially planned to use a phacoemulsification approach when feasible; however, in younger patients with soft lenses, manual aspiration was performed instead. In two cases, due to extensive capsular rupture, the procedure was converted to extracapsular cataract extraction. The placement and position of the intraocular lens (IOL) were documented for all cases. IOL power was calculated using optical biometry (Lenstar LS 900, Haag-Streit, Köniz, Switzerland) in cooperative patients, while contact ultrasound biometry (Axis Nano Biometer, Quantel Medical, Cournon d'Auvergne, France) was used for uncooperative patients. In cases with corneal opacity or irregularity precluding accurate keratometry, values from the fellow eye were used when appropriate.

In patients with adequate capsular support, a one-piece hydrophobic acrylic IOL (SA60AT, Alcon Laboratories Inc., Fort Worth, TX, USA) was implanted in the capsular bag. When posterior capsule rupture or zonular weakness precluded in-the-bag fixation, whereas sulcus support was intact, a three-piece hydrophobic acrylic IOL (MA60AC, Alcon Laboratories Inc., Fort Worth, TX, USA) was placed in the ciliary sulcus. In eyes lacking sufficient posterior support, an iris-claw anterior chamber IOL (Artisan Aphakia, Ophtec BV, Groningen, the Netherlands) was implanted and fixated to the anterior iris surface.

Target refraction was determined by age as follows: In children ≤ 1 year old, a target refraction of +7.00 diopters (D) of hyperopia was set. For children between 1 and 3 years old, the target was +5.00 D; for 3 to 5 years old, +3.50 D; for 5 to 7 years old, +2.50 D; for 7 to 9 years old, +1.50 D; for 9 to 10 years old, +1.00 D; and for those above 10 years old, plano was the refraction target for IOL selection.

At 4 weeks postoperatively, manifest refraction was performed on the operated eye, and cycloplegic refraction on the fellow eye. Based on these results, appropriate spectacle correction was prescribed. All pediatric patients received amblyopia therapy as clinically indicated.

### Statistical Analysis

Statistical analysis was performed using SPSS version 25.0 (IBM Corp., Armonk, NY, USA). Categorical variables were expressed as frequencies and percentages, and compared using the Chi-square or Fisher's exact test, as appropriate. Continuous variables were presented as means ± standard deviation (or median and interquartile range when not normally distributed) and compared using independent samples *t*-tests or Mann–Whitney U tests, as appropriate. Correlation between continuous variables was assessed using Pearson or Spearman correlation coefficients based on data distribution. A *P*-value of < 0.05 was considered statistically significant.

Missing data were handled using complete-case analysis; cases with missing critical information preventing accurate assessment were excluded from the relevant studies. No statistical imputation was performed for missing data.

##  RESULTS 

Of the 74 patients initially evaluated, 10 were excluded due to complications such as glaucoma (*n* = 4), retinal damage (*n* = 3), or other causes leading to additional surgical interventions or final visual acuity of LP or worse (*n* = 3). Consequently, 64 eyes from 64 patients were included in the final analysis. The mean age of the participants was 25.52 ± 22.00 years.

Among the 64 patients, 71.9% were male. Most patients (62.5%) were older than 10 years at the time of surgery. Seventeen patients (26.6%) were under 7 years old at the time of trauma. Open globe injuries accounted for the majority (75%) of cases, and sharp metal objects were the most frequent cause of injury.

Initial VA was better than hand motion in 52.5% of eyes. Most injuries occurred in Zone 1 (cornea). Vitreous prolapse was observed in 23.4% of cases. IOL placement varied, with 60.9% of lenses implanted in the capsular bag and 25.3% in the sulcus. Table 1 presents the qualitative factors potentially affecting final VA across different subgroups. No significant association was observed between final VA and sex, primary VA, injury zone, IOL location, or final refractive error.

However, patient age, injury type, and vitreous prolapse demonstrated statistically significant relationships with final VA (*P* < 0.05). Specifically, patients younger than 10 years showed better visual outcomes compared to older patients. Those with open globe injuries and without vitreous prolapse achieved higher final visual acuity.

Table 2 summarizes the quantitative factors influencing final VA. Among these, the size of the rupture was significantly correlated with visual outcome (*r* = 0.327, *P* = 0.024). No significant correlations were found for follow-up duration, time to surgery, or myopic shift in children under 10 years of age.

**Table 1:** Potential qualitative factors affecting the final visual acuity in different subgroups

**Characteristic**		**Number**	**Final visual acuity**	***P*** -value ${}^{*}$ * *
Sex		64		0.471
	Male	46	0.59 ± 0.54	
	Female	18	0.71 ± 0.54	
Age (years)		64		0.021
	< 10	24	0.8 ± 0.11	
	> 10	40	0.45 ± 0.06	
Primary visual acuity		61		0.338
	Hand motion or less than hand motion	32	0.54 ± 0.51	
	More than hand motion	29	0.64 ± 0.53	
Injury type		64		0.025
	Open	48	0.71 ± 0.46	
	Closed	16	0.37 ± 0.55	
Vitreous prolapse		64		0.012
	Yes	15	1.36 ± 0.71	
	No	49	0.51 ± 0.42	
Etiology		60		
	Sharp metal	23	0.72 ± 0.57	
	Wood	10	0.69 ± 0.53	
	Blunt trauma	14	0.44 ± 0.54	
	Stone	6	0.48 ± 0.41	
	Others	5	0.90 ± 0.41	
	Unknown	2	0.42 ± 0.51	
Injury zone		48		0.077
	Zone 1	36	0.62 ± 0.47	
	Zone 2	9	1.03 ± 0.47	
	Zone 3	3	0.71 ± 1.10	
Intraocular lens location		64		0.138
	In capsular bag	39	0.49 ± 0.43	
	In sulcus	16	0.80 ± 0.70	
	In anterior chamber	5	0.85 ± 0.71	
	Aphakia	2	1.10 ± 0.91	
	Fixation to sclera	2	1.25 ± 0.98	
Final refractive error (Diopters)		23		0.392
	< 1	11	0.54 ± 0.33	
	1-2	6	1.03 ± 0.72	
	2-3	6	0.83 ± 0.65	
${}^{*}$ Based on Chi-square.				

**Table 2:** Potential quantitative factors affecting the final visual acuity in different subgroups

**Characteristic**		**Mean ± SD**	**Correlation coefficient**	***P*** -value ${}^{*}$
Size of rupture (mm)		4.31 ± 2.3	0.327	0.024
Follow up (years)		5.66 ± 4.2	0.57	0.656
Time to surgery				
	Primary repair for open globe injuries (days)	1.81 ± 1.68	–0.179	0.547
	Cataract surgery in open globe injuries (weeks)	4.98 ± 3.21	0.083	0.569
	Cataract surgery in close globe injuries (weeks)	11.47 ± 5.50	0.31	0.056
Myopic shift in children under 10 years (diopters)		3.15 ± 2.1	0.148	0.511
SD, standard deviation				

##  DISCUSSION 

### Demographic Features

This study included 64 patients with a male predominance, consistent with previous studies reporting a higher incidence of ocular trauma among males. This trend indicates the greater exposure of men—particularly younger individuals—to occupational and recreational hazards.^[8,9,10,11]^ Thakur et al observed a similar gender disparity in their study on visual outcomes following traumatic cataract.^[12]^ These findings emphasize the need for targeted safety education and preventive strategies for high-risk groups.

Regarding age distribution, a substantial proportion of cases occurred in children under 10 years of age, who demonstrated poorer final visual outcomes compared with older patients. This contrasts with the findings of Ishfaq Ahmad et al, who reported better visual results in younger individuals.^[13]^ Several factors may explain the poorer pediatric outcomes, including ongoing ocular development, higher susceptibility to postoperative complications such as rapid posterior capsule opacification or secondary glaucoma, and the impact of amblyopia.

Amblyopia remains a major challenge in pediatric ocular trauma, especially when dense cataract or media opacity persists during critical stages of visual maturation. Even with timely surgical intervention, visual potential may be limited if amblyopia therapy is delayed or compliance is poor.^[14]^ Notably, 17 of our patients (26.6%) were younger than 7 years at the time of trauma, which corresponds to the critical period for visual development. The suboptimal outcomes observed in this group may therefore be partially explained by amblyopia rather than by surgical or structural factors alone. Given the high proportion of young children in our series, these findings highlight the importance of surgical planning, early visual rehabilitation, and long-term follow-up.

### Preoperative Factors

In this study, 52.5% of patients presented with initial VA better than hand motion. However, no statistically significant correlation was observed between initial and final VA (*P *= 0.338). While studies like Kuhn et al's ocular trauma score found the initial visual acuity as a significant factor for final outcomes, Shah et al reported no significant association between initial and final visual acuity, consistent with our findings.^[8,24]^

Most cases were open globe injuries, similar to rates reported in prior literature.^[8,15,16]^ Visual outcomes were significantly worse in open injuries compared to closed injuries, which could be attributed to the more complex nature of open injuries and their need for multiple surgical interventions.^[17]^ Snyder et al^[18]^ reported that 78.6% of traumatic injuries were open. However, Gogate et al^[19]^ found a higher incidence of traumatic cataracts in closed trauma cases (48.8%) compared to open injuries (39%).

Discrepancies in visual outcomes across studies are notable. While Shah et al^[7]^ and Smith et al^[20]^ reported better visual acuity in open injuries among adults, Shah et al's study of children with a mean age of 10.1 years found better outcomes in the closed trauma group.^[21]^ These variations may be due to differences in injury severity, patient demographics, socioeconomic factors, regional healthcare quality, and the surgical techniques employed. These findings highlight the need for individualized treatment strategies and consideration of contextual factors in managing traumatic cataract.^[15]^

The etiology profile in our series, with sharp metal as the leading cause, is in line with previous reports,^[22]^ though other studies have identified blunt trauma as more common.^[7,17]^

### Wound Features

Zone 1 injuries were the most frequent, aligning with Shah et al's report that 90.1% of injuries in their study of 1076 eyes occurred in this zone.^[21]^ Although there was no statistically significant difference in final visual acuity between the zones (*P* = 0.077), the *P*-value supports a potential trend, as prior literature suggests that posterior zone involvement carries a worse prognosis.^[3,15,23]^ The relatively low number of Zone-3 injuries in our study can be explained by two factors: first, such injuries are less common overall; second, Zone-3 injuries are often associated with severe posterior segment complications, including retinal damage and secondary glaucoma, which were excluded from our study. As a result, patients with Zone-3 injuries who developed these complications were not enrolled, while those without such complications were included.

Injury size showed a meaningful relationship with visual prognosis, consistent with Fujikawa et al, who found that larger wounds were associated with poorer outcomes. ${}^{\text{[28]}}$

### Intraoperative Factors

Consistent with previous research, we found no significant correlation between the timing of primary or secondary cataract surgery and final visual acuity. Gurung et al^[20]^ similarly reported no difference in visual outcomes between patients presenting within 1 month and those presenting years later.^[17]^ However, Saber Mohammad et al identified delayed presentation as a factor impacting visual acuity, highlighting the need for further investigation. ${}^{\text{[29]}}$

Vitreous prolapse was linked to worse vision. This decrease may be secondary to complications such as inflammation, delayed wound healing, and increased intraocular pressure. Traumatic cataract surgery, due to its complex nature and potential complications such as capsular tears, zonular dialysis, and vitreous prolapse, requires a personalized approach and careful clinical decision-making.^[24]^ Serna-Ojeda et al reported a vitreous prolapse incidence of 21.2%, which aligns with our findings. However, other studies have documented a higher incidence, up to 45%.^[16,24]^

IOL implantation was feasible in nearly all cases, with placement most often in the capsular bag.^[19,25]^ Patients with IOLs implanted in the capsular bag demonstrated better visual outcomes compared to those with sulcus or anterior chamber placement; however, this difference was not statistically significant. These results are consistent with those reported by Günayd ı n et al^[22]^ and other studies indicating that the site of IOL placement does not significantly influence final visual acuity.^[6,26]^ Conversely, Serna-Ojeda et al identified IOL placement as a significant factor affecting visual outcomes, with in-the-bag IOLs yielding the best results. It is important to note that the proportion of patients with in-the-bag IOLs was higher in their study, and reasons for alternative placements, such as complications preventing capsular bag fixation, were not analyzed.^[16]^

### Follow-up Duration and Outcomes

Among children under 10 years, most achieved acceptable refractive outcomes after surgery, though neither final refractive error nor myopic shift showed a strong relationship with vision. These results are comparable to those reported by Panahibazaz et al, emphasizing the benefit of age-adjusted IOL targeting in reducing reoperations.^[27]^

The mean follow-up duration in our cohort was 5.41 years, substantially longer than reported in many adult traumatic cataract studies, ${}^{\text{[31]}}$ yet consistent with prior pediatric series. ${}^{\text{[32]}}$ Notably, follow-up length demonstrated no significant association with final visual acuity.

While the 5-year follow-up period represents a key strength of this study compared to shorter-term cohorts, its adequacy remains questionable for pediatric populations. Amblyogenic risks and delayed traumatic complications (e.g., glaucoma and retinal detachment) may manifest decades post-injury, particularly in children with developing visual systems. Our data underscore the necessity of extended surveillance protocols ( ≥ 10 years) in pediatric ocular trauma registries to capture these late sequelae.

### Study Limitations

As a retrospective study, causal inferences are limited. Because our institution is a tertiary referral center, we may have a series that overrepresents complex cases. The exclusion of patients with severe complications, while allowing focus on cataract-related outcomes, may introduce selection bias and overestimate prognosis. Combining different VA testing methods also presents potential measurement variability, though conversion to logMAR helped standardize comparisons. Future multicenter studies with larger cohorts and long-term follow-up are warranted to confirm these findings and explore new preventive and surgical approaches.

In summary, the study identified age, size of injury, vitreous prolapse, and type of injury (open or closed injury) as significant predictors of final visual acuity. We also found that the final VA is influenced by multiple factors rather than a single factor. This highlights the importance of comprehensive evaluations that consider all contributing factors and the use of multivariate models to achieve a more accurate understanding of visual prognosis.

##  Financial Support and Sponsorship

This study was supported by a grant number (IORC-0305) from Ahvaz Jundishapur University of Medical Sciences.

##  Conflicts of Interest

None.
